# Decommissioning retired hemodialysis machines in Dutch hospitals: strategies and sustainability considerations

**DOI:** 10.1093/ckj/sfaf389

**Published:** 2025-12-12

**Authors:** Vincent Peters, Niels Verhoeven, Wendy van der Valk, Dennis Hulsen, Karin Gerritsen, Dennis van der Schrier, Thijs de Graaf, Frank van der Sande, Bram Kamps, Wim de Jong, Constantijn Konings, Barend Schouten, Peter Kotanko, Len Usvyat, John Larkin

**Affiliations:** Department of Information Systems and Operations Management, Tilburg University, Tilburg, The Netherlands; Department of Information Systems and Operations Management, Tilburg University, Tilburg, The Netherlands; Department of Information Systems and Operations Management, Tilburg University, Tilburg, The Netherlands; Department of Medical Physics, Jeroen Bosch Ziekenhuis, ’s-Hertogenbosch, The Netherlands; Department of Nephrology, UMC Utrecht, Utrecht, The Netherlands; Department of Medical Technology, Spaarne Gasthuis, Haarlem, The Netherlands; Department of MIT Medical Technology, Nephrology, Maastricht UMC+, Maastricht, The Netherlands; Department of Internal Medicine, Division of Nephrology, Maastricht UMC+, Maastricht, The Netherlands; Department of ICMT – Medical Technology, Franciscus Gasthuis, Rotterdam, The Netherlands; Department of Medical Physics, Elisabeth-TweeSteden Ziekenhuis, Tilburg, The Netherlands; Department of Nephrology, Catharina Ziekenhuis Eindhoven, Eindhoven, The Netherlands; Department of Nephrology, RadboudUMC, Nijmegen, The Netherlands; Renal Research Institute, New York City, NY, USA; Renal Research Institute, New York City, NY, USA; Renal Research Institute, New York City, NY, USA

**Keywords:** decommissioning strategy, hemodiafiltration, hemodialysis, hemodialysis machine, sustainability

## Abstract

**Background:**

The decommissioning of hemodialysis machines, particularly in the context of transitioning from hemodialysis to hemodiafiltration, remains understudied despite its importance for sustainable healthcare. This study evaluates decommissioning strategies for hemodialysis machines used by Dutch hospitals, analyzing the economic, social and environmental consequences.

**Methods:**

A qualitative, exploratory study was conducted through semi-structured interviews with 15 professionals from 11 Dutch hospitals that retired hemodialysis machines. The analysis focused on understanding decommissioning strategies and their economic, social and environmental consequences.

**Results:**

Five decommissioning strategies were identified: disposal, donation, reuse, sale and recycling/trade-in. Substantial variability and limited formalization in these strategies were observed across and within hospitals. Economic consequences included repair costs, depreciation and resale value. Social consequences were important, yet typically secondary. Environmental consequences were recognized but rarely formalized, although indirect environmental benefits from economically driven repair activities were acknowledged.

**Conclusions:**

Decommissioning strategies for hemodialysis machines in Dutch hospitals do not use formalized guidelines and are still predominantly shaped by economic drivers. The recognition that each decommissioning strategy entails distinct economic, social and environmental consequences highlights the need for more balanced decision-making. By embedding sustainability principles into hospital policies and standardizing decommissioning procedures, hospitals can move toward more circular and responsible dialysis care.

KEY LEARNING POINTS **What was known:**

- Little research exists on the decommissioning processes of hemodialysis machines, despite their economic, social and environmental consequences.
- Medical equipment disposal often lacks standardized practices and guidelines, resulting in inconsistent and reactive approaches.
- Sustainability considerations in medical equipment lifecycle management remain underexplored, limiting hospitals’ ability to enhance their sustainability performance.

**This study adds:**

- Five decommissioning strategies across 11 Dutch hospitals, primarily considering economic consequences, with social and environmental consequences being secondary considerations.
- That formalized national and institutional guidelines are absent, hindering consistent and sustainable decommissioning practices.

**Potential impact:**

- Findings could inform hospital policies and guidelines, promoting sustainable decommissioning using a structured and standardized process.
- Findings may stimulate policy development to incorporate sustainability into medical equipment lifecycle management, potentially reducing healthcare’s environmental footprint.

## INTRODUCTION

Patients with renal failure require a renal replacement therapy to sustain life, such as hemodialysis (HD), hemodiafiltration (HDF), peritoneal dialysis or kidney transplantation. In-center HD and HDF are the primary modalities [1, 2]. Most patients in the world use HD, but HDF is increasingly being used as a growing body of evidence demonstrates its advantages in terms of patient outcomes [3, 4]. While aging is part of a machine’s life cycle, the introduction of HDF may lead to the retirement of older, still functioning, HD machines. The transition to new HD machines raises important questions about responsible and sustainable decommissioning of old HD machines, particularly given ongoing global disparities in access to kidney care [2].

In most hospitals in low- and middle-income countries (LMIC), access to dialysis remains limited due to financial and infrastructural constraints [2, 5, 6]. At the same time, hospitals in high-income countries such as the Netherlands and Germany regularly decommission HD machines due to economic and functional considerations [2], despite being technically operable and potentially reuseable. Current decommissioning practices of retired HD machines in hospitals are reported to be fragmented and undocumented [7], resulting in limited understanding of the considerations and decisions that guide the decommissioning process.

Once an HD machine is retired, there are several ways it can be disposed of: manufacturer take-back for resale or reconditioning, donation or sale from high-income countries to LMICs, resale and refurbishment, recycling/trade-in, disposal or

keeping for spare parts [8]. Given the resource-intensive nature of dialysis, particularly the high consumption of energy, water and single-use materials [9], it is important to pursue a “green dialysis” approach, wherein economic, social and environmental implications of decommissioning are considered [2, 7].

The Triple Bottom Line (TBL) framework [10] provides a useful framework to analyze which decommissioning strategy to use and when. TBL takes into account not only economic, but also social and environmental, considerations. The economic bottom line extends beyond final performance to using resources efficiently and responsibly. For hospitals, this includes considering the residual value of medical equipment and realizing cost savings from waste prevention [11, 12]. The social bottom line contributes to the wellbeing of people. Hospitals can contribute by donating medical equipment and collaborating with other hospitals to enhance accessibility and availability of healthcare [13, 14]. The environmental bottom line relates to the organization’s need to minimize environmental impact—meeting present needs without compromising those of future generations [14, 15]. Hospitals can reduce their environmental impact through transparency in CO₂ emissions and waste management [14, 16]. Yet, little is known about the consequences of retiring HD machines, let alone whether and how hospitals consider these consequences when selecting decommissioning strategies.

This study evaluated decommissioning strategies for retired HD machines in Dutch hospitals and examined their economic, social and environmental consequences. By understanding how hospitals deal with retired HD machines, this study contributes to the development of more sustainable dialysis care provision that extends beyond medical walls.

## MATERIALS AND METHODS

Ethical approval was received from the Institutional Review Board (IRB) of the Tilburg School of Economics and Management (TiSEM) of Tilburg University (TiSEM-RP2058).

All participants gave oral and written informed consent in adherence with the Declaration of Helsinki. We followed two checklists for essential information to be included in qualitative research to warrant reliability and validity [17, 18].

### Design and setting

A qualitative, exploratory interview study was employed to enable in-depth understanding of decommissioning strategies of retired HD machines in Dutch hospitals.

### Recruitment

**Figure 1:**
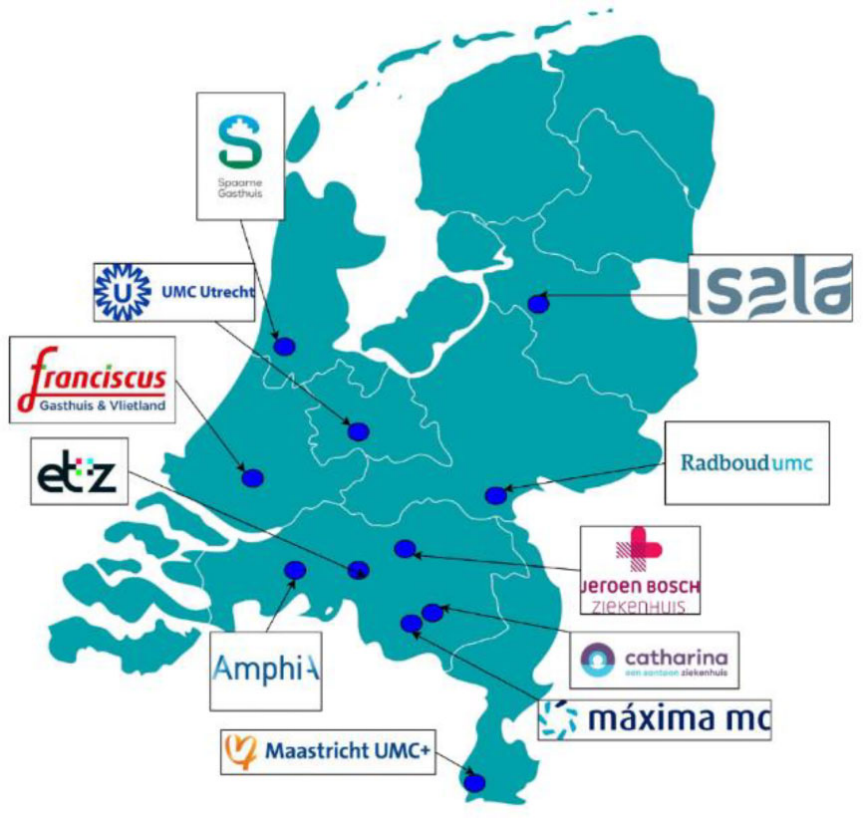
Overview of included dialysis centers.

In the Netherlands, 94 dialysis centers provide care for patients with renal failure, located at different hospitals and geographically dispersed over the country [19]. We used email and phone to contact the dialysis centers of 16 hospitals in the Netherlands. We aimed to select a range of dialysis centers varying in geographic locations and hospital type in order to select a representative set of participating centers. Based on this aim, we deliberately invited 16 out of the 94 centers to include in our research. They were chosen carefully, so that they demonstrated variety in the hospital type and geographic location, leading to a comprehensive view on retired HD machine decommissioning throughout the Netherlands. After repeated follow-up efforts, five dialysis centers indicated not having sufficient time for the interview. Eleven dialysis centers agreed to participate in this study (Fig. 1).

Utilizing purposive sampling, we asked the head of each dialysis center to identify participants with relevant knowledge and experience of the decommissioning of retired HD machines. This resulted in a total of 15 participants from 11 dialysis centers (Table 1). The dialysis nurses and internist-nephrologists staff the dialysis centers; the biomedical technicians, medical instrument technicians and medical physicists ensure the safe and efficient operation of HD machines through maintenance, quality control and technical oversight.

**Table 1:** Respondent characteristics.

Respondent number	Hospital number	Hospital type	No. of HD machines in clinic	Profession	Gender	Experience (years)	Interview duration (min)
1	A	Top clinical teaching hospital	33	Biomedical technician	Male	19	24
2	B	Top clinical teaching hospital	42	Biomedical technician	Male	37	24
3	B	Top clinical teaching hospital	42	Medical physicist	Male	11	24
4	C	University medical center	19	Internist-nephrologist	Female	15	43
5	C	University medical center	19	Dialysis nurse	Male	25	43
6	D	Top clinical teaching hospital	65	Biomedical technician	Male	3	25
7	E	Top clinical teaching hospital	35	Internist-nephrologist	Male	23	21
8	E	Top clinical teaching hospital	35	Medical instrument technician	Male	15	27
9	F	Top clinical teaching hospital	75	Biomedical technician	Male	2	32
10	G	Top clinical teaching hospital	30	Medical physicist	Male	30	36
11	H	Top clinical teaching hospital	39	Biomedical technician	Male	5	24
12	I	Top clinical teaching hospital	50	Medical instrument technician	Male	27	45
13	J	University medical center	36	Biomedical technician	Male	15	22
14	K	University medical center	25	Dialysis nurse	Male	17	30
15	K	University medical center	25	Medical instrument technician	Male	13	30

### Data collection

The semi-structured interviews were conducted in person between April and May 2025. We used literature to develop an interview protocol [7, 10], which included open-ended questions on the decommissioning process, and economic, social and environmental considerations (Supplementary data 1). Each interview was audio-recorded and transcribed verbatim, subsequently verified with the specific informant for the purpose of accuracy and resonance [20]. This did not result in any significant adjustments to the transcripts.

### Data analysis

Our thematic analysis [21] started with open coding by one researcher (N.V.) who reviewed two transcripts line-by-line. The codes were discussed among two researchers (N.V. and V.P.). Next, two researchers (N.V. and V.P.) coded the interviews independently and then compared and discussed their codes. During this process, initial codes were altered and new codes were added. Three researchers (N.V., V.P. and J.L.) discussed and assessed the outcomes of the coding until consensus was reached; this ensured investigator triangulation [22]. The remaining interviews were then coded by one researcher (N.V.) using the final version of the coding scheme (Supplementary data 2). Further data analysis was then performed in two steps. The first step involved identifying and analyzing the decommissioning strategies deployed by the individual hospitals. Open coding was employed to stay close to the data and allow concepts to emerge naturally, as opposed to imposing pre-conceived theoretical concepts. This resulted in the identification of five decommissioning strategies pursued by Dutch hospitals. The second step involved identifying economic, social and environmental considerations of decommissioning strategies using open coding. Based on those two steps, we were able to assign economic, social and environmental consequences to each decommissioning strategy.

## RESULTS

Studying 11 Dutch hospitals with an average number of 38.0 ± 15.7 HD machines (range 19–75 machines), we revealed substantial variation in HD machine decommissioning practices, few formalized procedures and minimal sustainability considerations. While hospitals follow a broadly similar technical process for taking HD machines out of service, the final decisions regarding retired HD machines differ depending on local policies, staff preferences and logistical feasibility. Successful identification of secondary use comes down to serendipity and just sheer luck: “I just happened to know a colleague in Poland, who I met at a conference, who could use our machine” (Respondent 3).

A foundational observation is that most hospitals lack a formalized strategy or policy for decommissioning of retired HD machines, including roles responsible for making decisions. Only one respondent reported the presence of a specific committee responsible for decommissioning of medical equipment: “This committee works according to [hospital] policy principles and determines what happens to the surplus equipment” (Respondent 8). This hospital had developed a structured decommissioning hierarchy: first considering trade-in while buying the new machine, followed by reuse of parts, sale or donation, and finally responsible disposal.

In contrast, most hospitals rely on *ad hoc* decisions based on the situation at hand. As Respondent 5 noted, “Nobody is concerned with that [decommissioning],” and decisions are typically made at the time of machine replacement without formal planning: “It is just looked at on the spot what the best solution is to get rid of them” (Respondent 4).

### Decommissioning strategies

The median age of HD machines was reported to be 4.25 years. One of 11 hospitals leased HD machines from the manufacturer, while the others purchased their HD machines. The median of the expected lifespan of the HD machines was 14.5 years. The factors driving recent decommissioning decisions were lifespan/operating hours (86% of machines) and a new tender (14% of machines).

**Figure 2:**
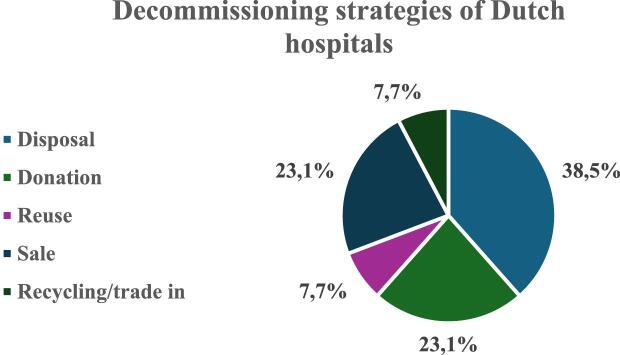
Identified decommissioning strategies of Dutch hospitals.

Various decommissioning strategies are employed, even within the same hospital, depending on the condition of the HD machines and available end-of-life opportunities. Among the 11 participating dialysis centers, we identified five decommissioning strategies: disposal (38.5% of centers), donation (23.1%), reuse (7.7%), sale (23.1%) and recycling/trade-in (7.7%) (Fig. 2).

Disposal is the most frequently applied strategy, particularly when equipment becomes unreliable or spare parts are no longer available. Respondent 4 mentioned: “They [hemodialysis machines] were so unreliable due to wear and tear that you do not do anyone any favors with them.” Similarly, Respondent 12 added: “Our machines are being removed because they are old and no longer maintained due to a lack of materials.” This is consistent with the primary reason for decommissioning observed, i.e. lifespan/operating hours.

Donation is widely regarded as a potential strategy when HD machines can be used further, especially for LMICs. Several respondents noted that retired HD machines often remain operational beyond the decommission date and could serve other healthcare institutions well. As Respondent 7 stated: “They do not break down so easily and you can easily use them second-hand again.” Others emphasized the cost advantages for recipient hospitals: “Because it might make a big difference in the costs they [LMICs] have to deal with” (Respondent 6). The rigid mechanical design of HD machines, with straightforward software and limited complexity, also supports their usability in LMICs: “They are quite suitable for donation and relatively easy to maintain there” (Respondent 8). Nonetheless, actual donation rates were less frequent than desired by respondents; while most respondents support the idea, they lack the network needed to arrange donations: “These are all questions that we would like the answer to.” Moreover, manufacturers do not typically facilitate second-hand use, with Respondent 7 noting: “I do not think the manufacturers have a lot of information about what they [manufacturer] should do with second-hand machines and how they [manufacturer] feel about it.”

Reuse of the machine, or parts of it, is technically possible and sometimes occurs informally between hospitals. As Respondent 1 explained: “I have a machine that will soon be decommissioned, and I will remove any parts I can reuse. Some components are quite expensive.” However, most hospitals refrain from reusing parts internally due to national policy or procedural limitations. For instance, Respondent 7 mentioned: “That [reusing parts] is not done. Any broken equipment is always replaced with new spare parts from the manufacturer.” There are exceptions, such as the reuse of functioning HD machines for internal research. One hospital repurposed a dialysis machine for lab use: “We are going to place one [dialysis machine] here in the lab for research” (Respondent 3).

Sale of HD machines is a strategy that is not often used. If HD machines retain sufficient market value, typically exceeding an internal threshold, it may be sold instead of donated: “We have the standard agreement that if a device can fetch €2000 or more, we choose to sell and are not allowed to donate because of hospital policy” (Respondent 8).

The recycling/trade-in strategy is not commonly pursued beyond the standard procedures for technical waste. HD machines are handed over to certified waste processors, who dismantle the machines only when economically viable. As one respondent stated: “The waste processor has a separation line and will dismantle it, but only if there is a business case for it” (Respondent 12).

### TBL of decommissioning

Our analysis revealed that decommissioning of HD machines by Dutch hospitals has economic, social and environmental consequences. While all three dimensions play a role, economic consequences were most prominent.

#### Economic consequences

Economic consequences are acknowledged most in each of the decommissioning strategies. High repair costs, expired depreciation cycles and discontinued technical support by manufacturers often mark the end-of-use: “It really is not worth it anymore to carry out additional maintenance” (Respondent 7). Procurement decisions similarly emphasized cost-efficiency, with hospitals seeking the best price-service balance and often preferring purchase over leasing: “But we said, it is better to buy them [hemodialysis machines] than to lease them” (Respondent 11). At end-of-life, resale was prioritized, with donation or part reuse considered only if financially viable: “Some parts are quite expensive, and I think it is a shame to throw them away” (Respondent 1). Even second-hand use was judged economically: “If that [second-hand sale] is economically justified and medically safe in from my point of view” (Respondent 5). In most instances, revenue from sales flowed into general hospital funds rather than dialysis centers, reinforcing the economic framing. Yet skepticism remained about resale prospects: “In the end, all these old hemodialysis machines will eventually end up on the scrap heap” (Respondent 1).

#### Social consequences

Social consequences seem less prominent, but nevertheless are an important result of decommissioning strategies. After economic consequences are addressed, input from nephrologists, nurses and technical staff may guide decisions, especially when new functionalities are involved: “Age is the primary driving factor here. The new features only come into play afterwards” (Respondent 10). Donation and reuse decisions often reflect the personal values and motivations of hospital staff: “I think it is a noble initiative to reuse the machine, so if that option were available, I would certainly support it” (Respondent 5), and “We really want to donate [to other countries] and help others [hospitals] with it as well” (Respondent 11). Respondents also expressed a desire for more alignment between hospitals: “I am actually very curious about how other dialysis centers manage this [decommissioning]” (Respondent 11). Some respondents highlighted the broader ethical and societal implications of disposal: “I see it much more broadly than just dialysis. Especially when it comes to sustainability, it is crucial to think about what happens to the products you dispose of: where you no longer have control over them” (Respondent 8).

#### Environmental consequences

Environmental consequences only have a limited role in decommissioning. There are no national guidelines or hospital-level policies specifically related to decommissioning medical equipment. However, technicians across all hospitals disclosed that ongoing repairs are a way to extend machine life. While repair is driven primarily by economic incentives, it also results in environmental benefits: “Since we handle maintenance ourselves, we have always kept the machines in excellent condition. When they reached 10 years of age, a component failed: the same issue we are now seeing in a 14-year-old machine. Thanks to our internal maintenance approach, we can continue using our machines much longer without needing to discard them” (Respondent 6). Several respondents argued to pay more attention to environmental considerations in future hospital policies: “It is more sustainable to keep the whole machine rather than just the parts.” (Respondent 8) and “I think we can still make considerable progress in terms of sustainability. It would be a shame to simply discard or dispose of everything” (Respondent 11).

### TBL consequences of decommissioning strategies

The analysis showed that each decommissioning strategy has economic, social and environmental consequences. A summary of the TBL consequences when selecting decommissioning strategies is presented in Table 2.

**Table 2:** Overview of TBL consequences of decommissioning strategies.

Decommissioning strategy	Economic consequences	Social consequences	Environmental consequences
Disposal	No financial return; possible disposal costs	Perceived waste; ethical discomfort	Potential landfill waste; resource loss
Donation	No financial return logistics cost	Solidarity; support for LMICs	Prolongs equipment use; prevents waste
Reuse	Potential cost saving on spare parts	Technical pride; efficient resource use	Extends machine lifespan
Sale	Financial return	Limited stakeholder involvement	Promotes second-hand use
Recycling/trade-in	Material value recovery	No strong stakeholder involvement	Recovers raw materials; avoids waste

## DISCUSSION

We explored decommissioning strategies for HD machines used in 11 Dutch hospitals and identified five distinct strategies: disposal, donation, reuse, sale and recycling/trade-in.

Our first key finding concerns the recognition that each decommissioning strategy entails economic, social and environmental consequences, and these consequences should be taken into account when opting for a strategy. However, our results also reveal that sustainability aspects are currently undervalued in decision-making. Hospitals could operate more sustainably if they did not focus primarily on economic consequences, but instead weighed social and environmental consequences more equally. Our findings show that economic consequences play a central role while decommissioning. Decisions to remove HD machines from service are predominantly triggered by factors such as high repair costs, completed depreciation cycles or a lack of manufacturer support for machines past their lifespan, confirming earlier research on economic viability as central to medical equipment lifecycle decisions [7]. Social consequences were important, yet typically secondary, shaped by professionals’ ethical views and a strong emphasis on donation and reuse to address healthcare disparities, mirroring previous findings on the role of healthcare professionals’ ethical perspectives and personal motivations in more operational decisions [13, 14]. Environmental consequences played only a minor role, reflecting the absence of sustainability guidelines in Dutch hospitals, consistent with broader calls for stronger integration of environmental criteria in healthcare [15, 23]. Scholars argue that sustainability should be embedded from procurement onward [9, 24], and that industry has a role in designing HD machines that are reusable, easily repaired and recyclable [7].

The second major finding is that sustainability only plays a limited role while decommissioning. Sustainable healthcare operations, including responsible decommissioning of medical equipment, have increasingly been recognized as vital due to the substantial environmental footprint of healthcare operations globally [23]; this is especially true for dialysis. HD is among the most resource-intensive therapies due to its heavy use of energy, water and single-use materials [9]. Reducing its environmental burden is therefore a global priority [25]. Our findings show that decommissioning strategies potentially contribute positively to sustainability objectives by extending equipment lifespan, reducing waste and recovering materials. However, the potential for recycling is low because of the chemical components and the fact that most pieces are a mixture of various materials [7]. Although donation is often seen as the most socially desirable option, its limited use reflects the complexity of this strategy rather than unwillingness by hospitals. High costs, lack of technical expertise in the receiving country, liability risks and increasing software-dependence make safe utilization in LMICs difficult. This highlights the need for collaboration between manufacturers, donors and recipients to make donation a feasible and sustainable strategy.

The third main result is related to the substantial variability in decommissioning strategies, with practices differing not only between but also within hospitals. Just one hospital had a formal decommissioning strategy in place. In the other 10 hospitals, decisions were made by a variety of professionals, and were typically based on the condition of HD machines and opportunities available at the end-of-life. There appears to be a need for formalized procedures and guidelines at institutional and national levels, given a lack of standardized protocols is known to contribute to inconsistent decommissioning outcomes and missed opportunities for improving sustainable practices [26].

Despite its merits, our study also has several limitations. First, our study only includes the perspectives of hospitals that retired HD machines, thereby excluding insights from dialysis machine manufacturers (e.g. Baxter, Fresenius Medical Care) and recipient hospitals. Future research could benefit from exploring suppliers’ decision-making processes. Similarly, recipient hospitals in LMICs should be studied, exploring whether and how their needs (could) inform decommissioning strategies of donor hospitals. Second, conducting in-person interviews may have introduced some degree of interviewer bias and socially desirable answers in participants’ responses. Also, participants’ answers may have been influenced by recollection bias, as their perceptions and memories could have evolved over time. We sought to minimize these limitations through using structured interview guide based on literature and by focusing questions on recent experiences. Last, an important question that arises from this study is: what would constitute an ideal decommissioning strategy? Given the interplay of economic, social and environmental consequences, there is no straightforward answer. We propose an ideal decommissioning strategy which could take the form of a circular path in which HD machines remain valuable beyond their initial use phase. After a period of clinical use and regular maintenance, dialysis centers could participate in early trade-in programs that allow suppliers to refurbish machines before they reach full end-of-life. These machines would then enter refurbishment departments—similar to, for example, Philips Medical’s refurbished systems program—where they could be upgraded or downgraded and recertified according to the desired safety and performance standards. Once refurbished, machines could be redistributed to LMICs, expanding access to dialysis care while reducing waste. Components/parts from machines no longer suitable for clinical use could be repurposed through a digital marketplace, enabling hospitals to exchange parts and share resources within a professional network. Ultimately, the remaining materials would undergo responsible recycling and recovery, ensuring that valuable resources re-enter the production chain. Throughout this process, continuous feedback and collaboration between hospitals, suppliers and professional societies would create a circular model for HD machines. Future research should not only outline the range of possible strategies but also explore how these could be operationalized in practice.

## CONCLUSIONS

This study provides in-depth insights into five decommissioning strategies for retired HD machines in Dutch hospitals: disposal, donation, reuse, sale and recycling/trade-in. Economic consequences are found to drive decommissioning practices, with opportunities to realize social and environmental benefits being underutilized. The absence of formal decommissioning policies and limited sustainability approaches highlight the need for standardized guidelines and institutional frameworks. Our findings offer insights for hospitals, policymakers and industry partners aiming to realize more sustainable and responsible decommissioning of medical equipment.

## Supplementary Material

sfaf389_Supplemental_Files

## Data Availability

The data that support the findings of this study are available from the corresponding author, V.P., upon reasonable request.
