# Supplementary material for: Decommissioning retired hemodialysis machines in Dutch hospitals: strategies and sustainability considerations
Source: Clin Kidney J. 2025 Dec 12;19(1):sfaf389. doi: 10.1093/ckj/sfaf389 (PMC12957914; doi:10.1093/ckj/sfaf389)
Supplement: sfaf389_Supplemental_Files [file sfaf389_Supplemental_Files.zip › Supplementary data 2. Coding scheme.docx]

**Supplementary data 2. Excerpt coding scheme**

| **Main code** | **Definition** | **Illustrative quote** | **Triple bottom line code** | **Definition** | **Illustrative quote** |
| --- | --- | --- | --- | --- | --- |
| Donation | Giving away used equipment to other healthcare facilities or organizations, often for free or as aid. | ‘*But the problem is that these countries [Low- and medium-income countries] often do not have the money to buy the line sets [Parts of machine]*.’ (Respondent 2)  ‘*That is also somewhat of a dilemma when it comes to donations to the third world [Low- and medium-income countries], for example. Donating something there, only for it to break down after two months and be impossible to repair.*’ (Respondent 8)  ‘*I think it's a noble initiative to donate that machine, so if that option were available, I would definitely participate.*’ (Respondent 5) ‘*Dialysis machines contain a lot of mechanical parts. So there are considerably fewer software components. These are fairly easy to donate and also easy to maintain there.*’ (Respondent 8) | Economic | Considerations related to financial costs, savings, or  economic benefits. | ‘*If it is economically viable and medically safe, I think donation is possible. But that is not the policy of this hospital.’* (Respondent 5) “*They should definitely do that if it's cheaper. Look ahead 10 years, when we have written them off financially. Are they still functional? They don't break down that quickly and you can easily use them again second-hand*.’ (Respondent 7)  ‘*So if it were to happen, when the 5008 [machine] was replaced, someone would come knocking on our door and say, well, we'll give you a little something [money] for it, and then they'll go to Angola [donation]. We thought about that carefully.*’ (Respondent 2) |
|  |  |  | Social | Considerations related to human or societal impact, such as patient well-being, ethical considerations, or public perception. | ‘*It may also be the case that someone within the organization, such as a nurse or someone else, is interested in sending the device to a charitable cause. Most input comes from the person themselves..*’ (Respondent 7)  ‘*I think that if you develop your own donation initiative and the Board of Directors likes the idea, then I think it could be arranged. Because I would say that it's also good for the hospital's reputation.*’ (Respondent 5)  ‘*That is also somewhat of a dilemma when it comes to donations to the third world, for example. Donating something there, only for it to break down after two months and be impossible to repair.*’ (Respondent 8) |
|  |  |  | Environmental | Considerations related to environmental impact, sustainability, emissions, and waste management. | ‘*A dialysis machine obviously needs a lot of disposables. In addition to disposables, you also have to take into account how much water it takes. One HDF online really costs liters and liters of water and are they [Low- and medium-income countries able to supply].*’ (Respondent 7)  ‘*It all depends on that supplier, how green you can have a machine, how green you can dispose of it, how green is the donation opportunity in terms of logistics, how green is the support towards third world countries [Low and medium income countries]?*’ Respondent 12) |
