# Supplementary material for: Decommissioning retired hemodialysis machines in Dutch hospitals: strategies and sustainability considerations
Source: Clin Kidney J. 2025 Dec 12;19(1):sfaf389. doi: 10.1093/ckj/sfaf389 (PMC12957914; doi:10.1093/ckj/sfaf389)
Supplement: sfaf389_Supplemental_Files [file sfaf389_Supplemental_Files.zip › Supplementary file 1. Interview questions.docx]

**Supplementary file 1. Interview questions**

**1. General introduction**

1. What is your position?
2. How many years have you been working in your current position?
3. How many years have you been working with dialysis machines?

**2. Introduction to decommissioning dialysis machines**

1. Who is your dialysis machine supplier?
2. Which dialysis machine(s) do you use?
3. What type of dialysis do you use?
4. What is the lifespan of a dialysis machine according to the manufacturer?
   1. What do you think is the actual lifespan in use?
5. How many dialysis machines do you have?
   1. How long have they been there?
6. What are the main reasons for decommissioning dialysis machines?
   1. Are you also replacing dialysis machines because of newer or more innovative dialysis machines?
7. Who decides when dialysis machines need to be replaced?
   1. Do these person(s) also determine what happens to the dialysis machines?
   2. What does the hospital do with retired dialysis machines?

**3. Decommissioning process**

1. What steps are required when decommissioning a dialysis machine?
   1. Do you always have the same way of dismantling?
2. How do you ensure the safe handling and disposal of medical waste associated with these dialysis machines?
3. What role does documentation play in the decommissioning process?

**4. Regulatory and compliance considerations**

1. What legal requirements apply to the decommissioning of dialysis machines?
2. Are there specific (environmental) regulations that hospitals must adhere to?

**5. Environmental and sustainability factors**

1. What are the environmental risks of disposing of dialysis machines?
2. Are there sustainable or environmentally friendly disposal methods available?
   1. If so, which ones?
   2. If not, is there a need for this?
3. Can parts of a dialysis machine be recycled or reused?
   1. If so, which ones? And do you make use of this?
   2. If not, why is this the case?

**6. Financial and logistical considerations**

1. What are the costs involved in decommissioning a single dialysis machine?
2. Are there any financial grants or programs available for recycling or donating retired dialysis machines?
3. How do you manage the logistics, such as transport and storage, during the dismantling and transport of retired dialysis machines?

**7. Alternatives to deletion**

1. Can retired dialysis machines be refurbished and reused in other healthcare settings?
   1. What options are there?
   2. What are the costs for this?
2. Are there any organizations that make it possible to donate retired, but functional dialysis machines to underdeveloped regions/countries?
   1. What are the costs to donate?
3. What factors determine whether a retired dialysis machine is suitable for donation or refurbishment?
4. Are and can retired dialysis machines be passed on to other healthcare institutions?
   1. If so, do you have any examples of this?
   2. If not, why is this not the case?
5. Can you exchange or return dialysis machines to your manufacturer?
   1. Do you have a collaboration with your supplier regarding sustainability aspects of your dialysis machine?
   2. Have you ever thought about dialysis machines as a service? That they are on loan?
   3. Would it be more advantageous for you if the manufacturer arranged the maintenance and replacement of the dialysis machine?
6. Have you or anyone else ever considered using a different strategy than the current tactic for retired dialysis machines?

**8. Insight into reuse**

1. Do you know what happened to the retired dialysis machines?
2. Do you or someone else influence the destination of retired dialysis machines?
   1. If so, in what way?

**9. Use of second-hand devices**

1. Does the hospital use second-hand dialysis machines?
   1. If so, how do you like this?
   2. If not, would you be interested in this?
2. Do you think there are possibilities in the use of second-hand dialysis machines?
   1. Or abroad?

**10. End of interview**

1. Do you have any additions that you expect to be important for this topic?
2. Do you have any questions?
